# How Experiences Affect Psychological Responses During Supervised Fasting: A Preliminary Study

**DOI:** 10.3389/fpsyg.2021.651760

**Published:** 2021-05-19

**Authors:** Qianying Ma, Chao Yang, Ruilin Wu, Manrui Wu, Wenjun Liu, Zhongquan Dai, Yinghui Li

**Affiliations:** ^1^Department of Psychology, Beihang University, Beijing, China; ^2^State Key Laboratory of Space Medicine Fundamentals and Application, China Astronaut Research and Training Center, Beijing, China; ^3^Beijing Ziyuan Fasting Training Center, Beijing, China

**Keywords:** fasting experience, mood states, stress, emergency, appetites

## Abstract

As an unusual event, fasting can induce strong physiological and psychological reactions, but there is still no clear understanding of how previous fasting experiences affect people’s responses to current fasting. This study aimed to investigate the influence of previous fasting experiences on participants’ basic physiological and psychological responses in a fasting experiment conducted under intensive medical monitoring. For a 22-day experiment divided into four phases (3-day Baseline; 10-day Complete Fasting; 4-day Calorie Restriction; and 5-day Recovery phases), a total of 13 persons participated; the participants were divided into a group with prior fasting experience (Experienced: *N* = 6) and a group without prior fasting experience (Newbie: *N* = 7). The results indicate no group differences in physiological responses (i.e., weight, glucose, and ketone bodies); however, differences in psychological states were observed, with the Newbie group showing more negative psychological states overall throughout the experiment (i.e., greater appetite, more negative mood states, more stress, less vitality, and fewer recovery resources). Hence, previous fasting experience may be a buffer against negative feelings during current fasting. For this reason, it is important to consider fasting experiences as a vital factor in future research.

## Introduction

Fasting, or the voluntary restriction of solid food consumption, has been practiced widely by different cultures and religions and used in clinical treatments for a variety of reasons (Kerndt et al., 1982; Venegas-Borsellino et al., 2018). Continuous calorie reductions and intermittent fasting, which involves cycles of fasting and eating periods, are common strategies for weight control and coping with overnutrition (Michalsen, 2010; Fond et al., 2013). Long-term complete fasting has been practiced as a clinical treatment, e.g., complete fasting for 10 days for patients with irritable bowel syndrome (Kanazawa and Fukudo, 2006) and even for 4 weeks fasting for euthyroid obese volunteers (Vagenakis et al., 1975). These fasting practices demonstrate that it is possible and safe for people to fast as long as an effective and suitable fasting protocol is in place.

There are consistent findings on physiological responses to fasting, such as weight loss and decreased fat mass index, across various fasting protocols (Kerndt et al., 1982; Varady, 2011). Additionally, research has shown a metabolic transition from utilizing glucose to ketones as the major cellular fuel source during fasting (see review, Phillips, 2019). Primary blood ketones can induce the transcription of brain-derived neurotrophic factors, which play critical roles in learning, memory, and the generation of new nerve cells in the hippocampus (Mattson et al., 2018; Phillips, 2019). This result indicates a potential role of fasting in benefiting neuron bioenergetics, plasticity, and resilience to stress and in maintaining or even enhancing cognitive performance (Longo and Mattson, 2014; Mattson et al., 2018; Phillips, 2019). From this perspective, a regular practice of appropriate fasting may contribute to extending the lifespan and healthspan (Phillips, 2019; Hwangbo et al., 2020).

Compared to consistent findings for physiological responses, there is less consistency in psychological responses to fasting. First, coincident with people’s intuitive anticipations of food deprivation, research has found that short-term or intermittent fasting could induce irritability and negative mood states (Bolton et al., 2014; Solianik et al., 2016; Nugraha et al., 2017) as well as subjective feelings of sleepiness and fatigue (Roky et al., 2003; Alkandari et al., 2012). In contrast, some research has reported that mood states remain stable during short-term calorie deprivation or Ramadan fasting (Lieberman et al., 2008; Solianik and Sujeta, 2018) and that fasting does not significantly influence fatigue and sleepiness (Bahammam et al., 2013; Nugraha et al., 2017). In addition, research has found that fasting can result in positive experiences (e.g., achievement, pride, and control after 18 h of fasting Watkins and Serpell, 2016) and decreased negative mood states after practicing a 3-month calorie restricted diet, which is termed the mood enhancement phenomenon (Michalsen, 2010; Fond et al., 2013; Hussin et al., 2013).

The contradictions between these results concerning the effect of fasting on psychological responses may be the result of varying fasting protocols and different participant groups. Previous studies have used various types of fasting designs: calorie restriction (Hussin et al., 2013), intermittent fasting (e.g., alternative day fasting, Varady et al., 2013), Ramadan fasting (Nugraha et al., 2017), and continuous complete fasting (e.g., 18 h, Watkins and Serpell, 2016; 2 days, Solianik and Sujeta, 2018; and 10 days, Kanazawa and Fukudo, 2006). Participant groups have also varied in age (young, e.g., 26 years old: Nugraha et al., 2017; and older, e.g., 59 years old: Hussin et al., 2013), gender (female: Watkins and Serpell, 2016; male: Nugraha et al., 2017; and mixed gender: Uher et al., 2006) and health status (healthy participants: Watkins and Serpell, 2016; and patients: Kanazawa and Fukudo, 2006; Michalsen, 2010; Fond et al., 2013).

In addition to varying experimental protocols, many factors may affect psychological responses. First, motivations and expectations of fasting may play a critical role in psychological responses. For example, in various religions, fasting is considered a means to fortify the body, purify the spirit, and elevate consciousness (Venegas-Borsellino et al., 2018). Additionally, fasting is considered a self-empowering, cost-free strategy of weight management (Phillips, 2019). Hence, fasting may be experienced as pleasurable and tolerated by people who value their religion or aim to lose weight. In contrast, fasting may generate negative mood states for those who do not have religious and weight concerns. Second, fasting is closely related to self-control (Gailliot and Baumeister, 2007). On the one hand, fasting is a process requiring considerable cognitive effort, including self-control (e.g., controlling desires to eat and maintaining fasting protocols for several days; Finlayson et al., 2007). On the other hand, successfully completing periods of fasting may increase feelings of self-control (Moreno-Domínguez et al., 2012; Watkins and Serpell, 2016). Third, the initial baseline of psychological states is important to consider. For example, participants may report decreased mood states if their mood states, especially their positive moods, are generally high and vice versa.

Other than the factors mentioned above, another factor that may affect psychological responses is previous fasting experience. People who have fasted before may have an understanding of how fasting feels. In contrast, people who have never fasted before will encounter their first experience of a lack of food during an experiment. However, fasting experience is often ignored in participant selection and in final reports. One study considered whether participants had previous fasting experience before they began an 18-h fasting experiment. However, researchers have focused on differences between fasting and non-fasting and have not reported the potential differences caused by fasting experience (Watkins and Serpell, 2016). Alternatively, fasting experience is controlled as an irrelevant variable (e.g., participants “have fasted during Ramadan at least once before”; Nugraha et al., 2017), but this control is still rare in research.

Hence, the current study examined the effect of previous fasting experiences on basic physiological and psychological responses in healthy individuals. We conducted a 22-day fasting experiment under intensive medical monitoring, which was divided into 4 sessions: 3-day Baseline, 10-day Complete Fasting, 4-day Calorie Restriction, and 5-day Recovery sessions. We hypothesized that during the 22-day experiment, all participants would have similar basic physiological reactions but that participants who did not have previous fasting experience would report stronger negative feelings than those who had fasting experience.

It should be clarified that the aim of this study was not to recommend long-term complete fasting (e.g., 10 days without any solid food intake) as a routine practice in daily life, especially without intensive medical monitoring. What we wanted to investigate here is how previous experience affects participants’ responses to fasting and how this information could enrich future research on fasting and eating behaviors.

## Materials and Methods

### Participants

Participants were recruited through online advertisements. To eliminate potential gender differences in fasting reactions, the post requested that only males participate in the 22-day experiment. A total of 60 interested participants responded and were invited for further screening. Of these respondents, 44 participants dropped out during basic screening due to age, weight, BMI, underlying health conditions or religious beliefs. After screening, 16 participants were deemed suitable to voluntarily participate in the experiment, and three dropped out for personal reasons before the start of the experiment (e.g., schedule conflicts). A total of 13 participants (all male; aged between 28 and 55 years with a mean age of 40 ± 8 years) completed the entire experiment.

The inclusion criteria are as follows: BMI of between 18 and 35, weight of between 60 and 90 kg, and height of between 165 and 190 cm. All inclusion criteria were confirmed before the experiment. The exclusion criteria applied are as follows: history of an eating disorder or any chronic illness, diabetes mellitus, history of cancer, history of cardiovascular disease, history of metabolic diseases, or tobacco or alcohol dependence. All exclusion criteria were assessed *via* a complete medical examination at Longgang Central Hospital in Shenzhen, China. Individuals who had fasted in the past 3 months prior to recruitment were also excluded from the study due to safety considerations.

The study was approved by the Ethics Committee of the Space Institute of Southern China. All participants gave their written informed consent to participate in the study. To minimize the risks of fasting, such as hypoglycemia, participants were given advice and information on fasting and its possible side effects. The participants were also informed to stop fasting immediately if they felt extreme discomfort and to report such symptoms to doctors and researchers who also stayed at the Space Institute of Southern China during the experiment.

Previous fasting experience was assessed by the participants’ self-reports during the screening stage. The participants were asked whether they had practiced fasting before. Participants reporting that they had fasted at least once before the experiment were categorized as “the Experienced group.” Participants reporting that they would be practicing fasting for the first time were categorized as “the Newbie group.” Six of the participants had fasting experience (43 ± 9 years) and seven did not (37 ± 7 years).

### Experimental Protocol

All participants arrived at the laboratory at the Space Institute of Southern China 3 days before the experiment started. In these 3 days, participants received detailed information about the experimental aims, procedure, time schedule, and laboratory settings.

**Figure 1:**
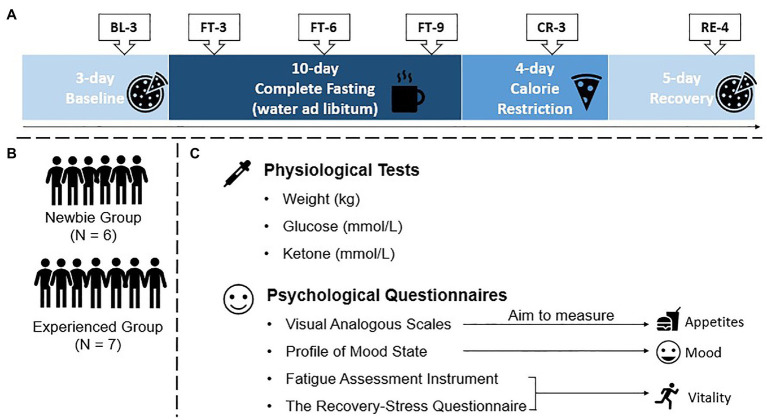
Protocol of the 22-day experiment, test points, groups, and materials. **(A)** Protocol and test points (BL-3: 3rd day of Baseline; FT-3, FT-6, FT-9: 3rd, 6th, and 9th days of Complete Fasting, respectively; CR-3: 3rd day of Calorie Restriction; and RE-4: 4th day of Recovery). Participants were subject to no food intake limitations in the Baseline and Recovery sessions, they could drink water without solid food intake during the Complete Fasting session, and they could obtain limited food from the researchers during the Calorie Restriction session. **(B)** Two groups involved in the current experiment. **(C)** Physiological and psychological tests used in the current experiment for all six test points.

The whole experiment lasted 22 days and involved four sessions: Baseline, Complete Fasting, Calorie Restriction, and Recovery sessions (Figure 1A). In the 3-day Baseline session, there was no limitation on food intake. Participants could go to the restaurant at the institute for their daily meals, and they were free to order any food provided by restaurants. There was one test point on the third day of this session (BL-3), which was considered as the baseline test. We applied the Baseline session to let the participants become familiar with the laboratory environment.

In the Complete Fasting session, participants were subjected to 10 days of fasting. During complete fasting, participants could not eat anything but could drink water at any time they wanted, and they were encouraged to do so to avoid dehydration during fasting. There were three test points during the Complete Fasting session (FT-3, FT-6, and FT-9), illustrating a trajectory of how the participants reacted to complete fasting.

After the Complete Fasting session, the Calorie Restriction session occurred. We applied this session to avoid potential negative effects from sudden eating, and participants received daily meals from the researchers only. The meals had fewer calories than the participants’ normal daily energy intake, and the quantity of food gradually increased during the Calorie Restriction session, which provided an opportunity for their bodies to gradually readapt to their normal food intake. One test point was applied during the Calorie Restriction session (CR-3).

The final session was the Recovery session. The participants were allowed to eat food at any time (e.g., snacks), and their daily meals were provided by the restaurant at the Space Institute of Southern China. There was one test point during the Recovery session (RE-4) and another medical examination at Longgang Central Hospital was conducted on the same day. We set this session to allow the participants have time to return to their normal food intake. The medical examination revealed that no participants showed physical side effects after fasting.

The fasting experiment required intensive monitoring of the participants’ physical states because the participants had to avoid any solid food intake. Therefore, the participants stayed in designated areas of the institute for the entire duration of the 22-day experiment. During the experiment, with the exception of physiological and psychological tests, participants did not receive any work requirements from the experimenters, and they could perform their own work through network telecommuting. The Experienced and Newbie groups were mixed during the experiment (Figure 1B). To avoid potential social desirability bias, the participants were not told that there were two groups based on fasting experience.

### Measurements and Materials

#### Physiological Measurement

The following physiological variables were assessed: weight, glucose, and blood ketones (Figure 1C). Blood samples were collected at 9 a.m. and then centrifuged at 3,000 *g* for 10 min. Serum was collected and stored at −70°C until assay. Glucose was measured by a blood automatic analyzer (Cobas8000 c701, Roche, Switzerland) housed at Longgang Hospital. Ketones were measured by blood test strips (blood β-ketone test strips, Abbott, America).

#### Psychological Measurement

Questionnaires were used to assess the participants’ psychological responses to the 22-day fasting experiment (Figure 1C). A visual analog scale was used to measure the participants’ appetites, which were directly affected by fasting. We also used the Profile of Mood States to assess the mood states of the participants and the Fatigue Assessment Instrument and Recovery-Stress Questionnaire to measure the participants’ vitality during the whole experiment.

*The visual analog scale* (*VAS*) was used to assess appetites. Hunger, a desire to eat and fullness were assessed by a 100-mm visual analog scale (Wewers and Lowe, 1990) ranging from 0 (“I am not hungry at all/I do not want to eat at all/I am not full at all) to 9 (“I am extremely hungry/I want to eat badly/I am totally full).

*The Profile of Mood State* (*POMS*) was used to assess the mood states of the participants (Wang et al., 2014). The 65-item instrument is used to measure six domains of mood states, including Tension-Anxiety, Depression-Dejection, Anger-Hostility, Vigor-Activity, Fatigue-Inertia, and Confusion-Bewilderment. The questionnaire is answered on a five-point scale ranging from 1 (not at all) to 5 (extremely).

The *Fatigue Assessment Instrument* (*FAI*) includes 29 items (Schwartz et al., 1993). There are four dimensions of fatigue: severity, situation specificity, psychological consequences, and response to rest/sleep. Responses for each item were measured on a seven-point Likert scale ranging from 1 (“Completely disagree”) to 7 (“Completely agree”). Higher scores for severity, situation specificity, and psychological consequences indicate a higher level of fatigue. Higher scores for response to rest/sleep indicate a lower level of fatigue.

The *Recovery-Stress Questionnaire* (*RESTQ*) was used to assess the balance between psychological stress and recovery states in individual, social, emotional, and physical dimensions (Yuan et al., 2019). The total stress score is the mean of scores for seven stress subscales (General Stress, Emotional Stress, Social Stress, Conflicts/Pressure, Fatigue, Lack of Energy, and Somatic Complaints). The total recovery score is the mean of scores for five recovery subscales (Success, Social Relaxation, Somatic Relaxation, General Well-Being, and Sleep Quality).

The Chinese versions of these questionnaires were applied in the current study. While the sample size used in this study was too small to estimate internal consistency and test-retest reliability, studies conducted on larger Chinese sample sizes have shown that these questionnaires have acceptable Cronbach’s alpha coefficients and test-retest reliabilities.

### Statistical Analysis

A previous study with similar experimental conditions as ours involved nine participants who completed 10 days of fasting (Fazeli et al., 2015). However, due to safety considerations, we still chose to use a relatively small sample rather than recruiting a large sample directly. All participants were carefully screened for their health status, and the experiment was conducted in a strictly controlled laboratory environment under doctors’ supervision. To avoid bias as much as possible, we used the nonparametric Mann-Whitney U-test. The Mann-Whitney U-test does not carry any assumptions about the distribution of data but tests whether two independent samples have been drawn from a population with the same distribution (Corder and Foreman, 2009), which is suitable for the small sample size used in the present study.

All Mann-Whitney U statistical analyses were performed using SPSS 21 (IBM SPSS, 2010, Chicago, IL, United States). Two-tailed statistical significance was determined as *p* < 0.05. Descriptive statistics (means and SDs) were calculated for all physiological and psychological variables. Because SPSS 21 does not provide effect size analysis for the Mann-Whitney U-test, the *Cohen’s d* effect size was further calculated with an online calculator when SPSS 21 revealed significant results.^1^

To identify the minimum effect that can be reliably detected with our sample size, we applied a sensitivity power analysis to our experiment using G*power 3.1.9.4 (Faul et al., 2009). For the Mann-Whitney test (two groups), the alpha significance criterion was set to 0.05, the standard power criterion was set to 80%, and the sizes of groups were set to 6 and 7 with two tails and Laplace parent distribution. The sensitivity power analysis shows that the minimum effect size *Cohen’s d* should be 1.35. As shown in the results section, the significant results of the current experiment meet this effect size requirement (for further details, see the Supplementary Tables S1–S5).

Moreover, a Bayes factor analysis was used to evaluate any effects found in the experiment (JASP0.14.1.0). Here, we calculated Bayes factors to interpret our main results. BF indicates the Bayes factor in favor of the alternative hypothesis (H1) over the null hypothesis (H0). For example, if BF = x, this means that the data are x times more likely to support the alternative hypothesis (e.g., BF_+0:_ the Newbie group had a more negative mood than the Experienced group) than the null hypothesis (e.g., there is no difference between the two groups). BF > 3 indicates substantial evidence to support the alternative hypothesis (van Doorn et al., 2020). The results of the Bayes factor analysis show that the significant results found in our preliminary experiment present substantial evidence to support the alternative hypothesis (i.e., the Newbie group experienced more negative states and fewer positive states than the Experienced group; for further details, see the Supplementary Tables S1–S5).

## Results

### Effect of Fasting Experiences on Physiological Responses

**Figure 2:**
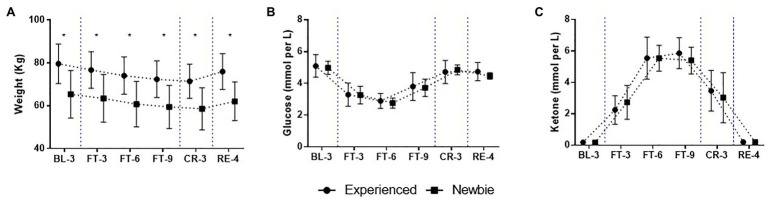
Mean and SD of physiological scores over the 22-day experiment for the Experienced and Newbie groups. **(A-C)** The scores of the three basic physiological variables. Symbols represent mean scores for two groups, and error bars represent the SD of the mean. ^*^p < 0.05 indicates significant differences between the two groups at that test point. Blue dashed lines indicate the different sessions.

The long-term fasting experiment had a strong physiological effect on the participants, and their physiological responses to fasting varied over time. Although the participants’ weights varied significantly, with those with fasting experience generally weighing more (Figure 2A), the pattern of weight loss between the two groups was comparable (all *p* < 0.05, all Cohen’s *d* > 1.48). Furthermore, there were no group differences in glucose and ketone levels (Figures 2B,C) Supplementary Table S1), and the increase in ketone bodies compensated for the decrease in glucose due to fasting, reaching a new metabolic balance at around FT-6.

### Effect of Fasting Experience on Psychological Responses

**Figure 3:**
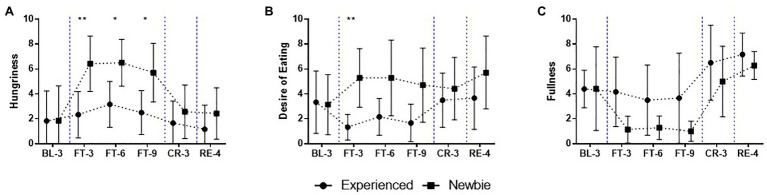
Mean and SD of appetite scores for the 22-day experiment for the Experienced and Newbie groups. **(A-C)** The scores of the subscales of visual analog scale. Symbols represent mean scores for the two groups, and error bars represent the SD of the mean. ^*^p < 0.05 and ^**^p < 0.01 indicate significant differences between the two groups at a given test point. Blue dashed lines indicate the different sessions.

**Figure 4:**
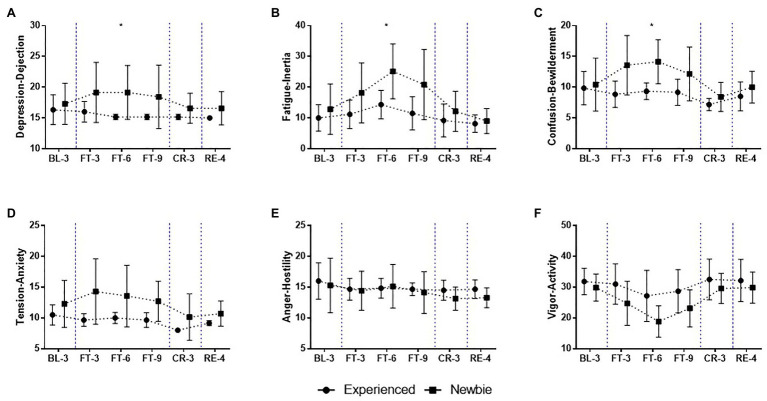
Mean and SD of mood state scores over the 22-day experiment for the Experienced and Newbie groups. **(A-F)** The scores of the subscales of the profile of mood states (POMS). Symbols represent mean scores for the two groups, and error bars represent the SD of the mean. ^*^p < 0.05 indicates significant differences between the two groups at a given test point. Blue dashed lines indicate the different sessions.

**Figure 5:**
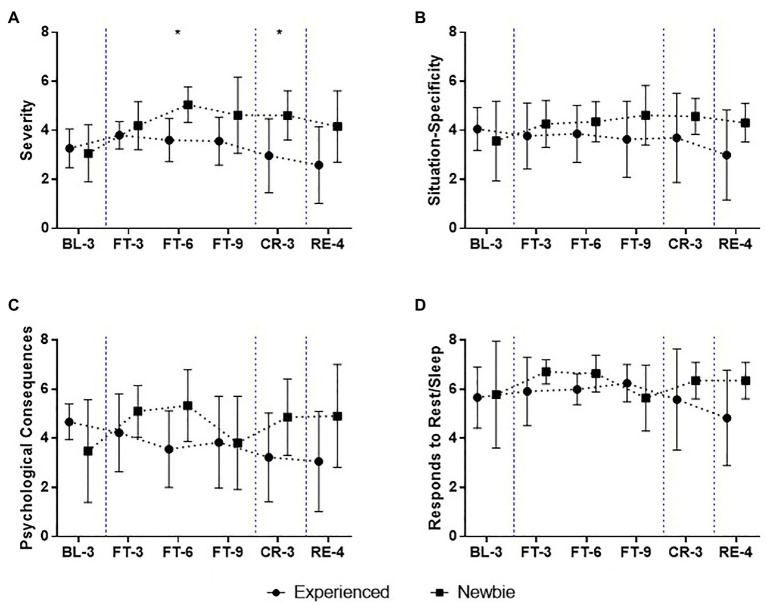
Mean and SD of fatigue scores over the 22-day experiment for the Experienced and Newbie groups. **(A-D)** The scores of the subscales of the fatigue assessment instrument (FAI). Symbols represent mean scores for the two groups, and error bars represent the SD of the mean. ^*^p < 0.05 indicates significant differences between the two groups at a given test point. Blue dashed lines indicate the different sessions.

**Figure 6:**
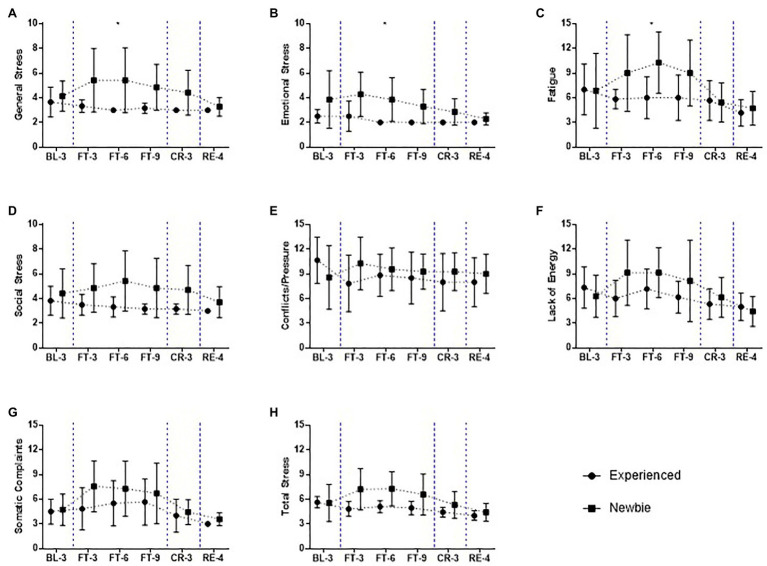
Mean and SD of subscales of stress scores over the 22-day experiment for the Experienced and Newbie groups. **(A-H)** The scores of the subscales related to the stress in the recovery-stress questionnaires (RESTQ). Symbols represent mean scores for the two groups, and error bars represent the SD of the mean. ^*^p < 0.05 indicates significant differences between the two groups at a given test point. Blue dashed lines indicate the different sessions.

Although all participants exhibited similar physiological responses, the questionnaire results reveal that the two groups significantly differed in psychological responses during the 22-day experiment (Figures 3–6), especially during the Complete Fasting session.

#### Effect of Fasting Experience on Appetite

Because feelings of hunger and fullness and the desire to eat were directly affected by food deprivation, changes in subjective appetite were a major concern during the fasting periods. Visual analog scales were used to measure the participants’ subjective appetite responses (Figure 3; Supplementary Table S2).

In the Baseline session (BL3), participants reported similar scores for hunger, the desire to eat and fullness between the two groups. During the Complete Fasting session, the Newbie group had a greater appetite than the Experienced group. Compared to those of the Experienced group, the Mann-Whitney U-test revealed significantly higher scores for Hungriness for the Newbies (FT-3: *M ± SD_Experienced_* = 2.33 ± 1.86, *M ± SD_Newbie_* = 6.43 ± 2.23, Mann-Whitney *U* = 3, *p* = 0.008, Cohen’s *d* = 2.04; FT-6: *M ± SD_Experienced_* = 3.17 ± 1.83, *M ± SD_Newbie_* = 6.50 ± 1.87, Mann-Whitney U = 3.5, *p* = 0.015, Cohen’s *d* = 1.93; FT-9: *M ± SD_Experienced_* = 2.5 ± 1.76, *M ± SD_Newbie_* = 5.71 ± 2.36, Mann-Whitney U = 6.5, *p* = 0.035, Cohen’s *d* = 1.40) and significantly higher scores for the desire to eat at the beginning of Complete Fasting session (FT-3: *M ± SD_Experienced_* = **1.33 ± 1.03**, *M ± SD_Newbie_* = **5.29 ± 2.36**, Mann-Whitney U = 1.5, *p* = 0.002, Cohen’s *d* = 2.43). Although other test points did not reveal significant results, Figure 3 shows that the inexperienced group had higher scores for hunger (Figure 3A), higher scores for the desire to eat (Figure 3B), and lower scores for fullness (Figure 3C).

In addition, different patterns were found between the groups during the Fasting session. For the Newbie group, the scores for hunger and the desire to eat sharply increased at the beginning of the Complete Fasting session (FT-3), and the scores slightly decreased but remained at a high level during the remainder of the Complete Fasting session. This result may suggest that the participants were unfamiliar with the experience of fasting at first and gradually adapted to the experience. In contrast, for the Experienced group, the scores for hunger and fullness remained relatively stable, and their desire to eat even decreased during the Complete Fasting session. After the participants had access to food, the inexperienced group’s scores for hunger decreased to the baseline level, and these participants reported a greater desire to eat than at baseline. For the Experienced group, the scores for hunger and the desire to eat returned to baseline levels. All of the participants reported high scores for fullness at CR-3 and RE-4.

#### Effect of Fasting Experience on Mood States

During the experiment, the Newbie group generally experienced more negative mood states and lower scores for vigor than the Experienced group as assessed by the POMS (Figure 4; Supplementary Table S3).

The two groups had similar mood states at baseline. The Newbie group reported an increase in negative mood states during complete fasting. In the middle of the Complete Fasting session (FT-6), the higher scores of negative moods for the Newbies reached significance for Depression-Dejection (Figure 4A; *M ± SD_Experienced_* = 15.17 ± 0.41, *M ± SD_Newbie_* = 19.14 ± 4.38, Mann-Whitney U = 4.5, *p* = 0.014, Cohen’s *d* = 1.73), Fatigue-Inertia (Figure 4B; *M ± SD_Experienced_* = 14.33 ± 4.63, *M ± SD_Newbie_* = 25.14 ± 8.91, Mann-Whitney U = 6.5, *p* = 0.035, Cohen’s *d* = 1.40), Confusion-Bewilderment (Figure 4C; *M ± SD_Experienced_* = 9.33 ± 1.37, *M ± SD_Newbie_* = 14.14 ± 3.58, Mann-Whitney U = 4, *p* = 0.014, Cohen’s *d* = 1.82). After obtaining food during the Calorie Restriction and Recovery sessions (CR-3, RE-4), all participants reported a decline in negative mood states, and the scores were even lower than their baseline levels.

Different trends in mood states were found between the two groups during the Complete Fasting session. For the Newbie group, Tension-Anxiety, and Depression-Dejection scores peaked at the start of fasting (FT-3; Figures 4A,D) and then gradually decreased but remained at a high level. The Newbie group also showed increases in Fatigue-Inertia and Confusion-Bewilderment scores, which peaked in the middle of the Complete Fasting session (FT-6, Figures 4B,C). Then, Fatigue-Inertia and Confusion-Bewilderment scores decreased at FT-9. Anger-Hostility scores decreased during fasting except for an increase at FT-6 (Figure 4E). The Experienced group reported lower and more stable scores for Depression-Dejection, Confusion-Bewilderment, Tension-Anxiety, and Anger-Hostility during the Complete Fasting session than during the Baseline session, and the scores of these negative mood states remained relatively stable (Figures 4A,C–E). The exception is the Fatigue-Inertia scores, which increased until FT-6 and then decreased to the baseline level (Figure 4B).

The trends for Vigor-Activity, which can be described as a positive mood state, were similar between the two groups (Figure 4F). The Vigor-Activity scores decreased when the Complete Fasting session began and then gradually increased after FT-6. The Newbie group reacted considerably, as indicated by lower scores for vigor during fasting. When all participants had access to food, the Vigor-Activity scores for both groups returned to baseline levels.

#### Effect of Fasting Experience on Vitality Responses

According to the Fatigue Assessment Instrument report, the Newbie group reported higher scores for fatigue overall than the Experienced group (Figure 5; Supplementary Table S4).

When the Complete Fasting session began, there was an increase in scores for severity, peaking at FT-6. The scores remained high but gradually decreased after FT-6. For the Experienced group, the scores for Severity were higher at FT-3 than during the Baseline session and then began to decrease. A significant difference in the severity of fatigue was observed at FT-6 (Figure 5A; *M ± SD_Experienced_* = 3.61 ± 0.88, *M ± SD_Newbie_* = 5.05 ± 0.73, Mann-Whitney U = 4, *p* = 0.014, Cohen’s *d* = 1.82), similar to the difference in mood states found for Fatigue-Inertia (Figure 4B). However, unlike fatigue, a sense of tiredness, a lack of energy or exhaustion continued, remaining as a significant group difference at CR-3 (Figure 5A; *M ± SD_Experienced_* = 2.97 ± 1.51, *M ± SD_Newbie_* = 4.61 ± 1, Mann-Whitney U = 6.5, *p* = 0.035, Cohen’s *d* = 1.40). The Newbie group reported high scores for fatigue at CR-3 and RE-4, but the Experienced group had low scores for fatigue (even lower than the Baseline) when they obtained food.

Although there were no significant group differences in terms of the sensitivity of fatigue to particular circumstances (heat, cold, stress; Figure 5B) and the loss of patience, motivation, or the ability to concentrate (Figure 5C), varying patterns of fatigue were found between the two groups. For the Newbie group, the scores for Situation Specificity and Psychological Consequences increased during fasting and remained high. In contrast, the Experienced group reported a decrease in fatigue during fasting, and the scores decreased to baseline levels during the Recovery session. Interestingly, the Newbie group also reported high Response to Rest/Sleep scores, except at FT-9 (Figure 5D), suggesting that fatigue could be reduced by rest. The Experienced group reported lower Response to Rest/Sleep scores, especially during the Calorie Restriction and Recovery sessions, showing that sleeping or resting did not reduce their fatigue.

The participants’ stress levels and current capabilities for recovery were measured by the Recovery-Stress Questionnaire (Figure 6; Supplementary Table S5). Overall, the Newbie group felt more stress, and they had fewer resources to recover from high-stress states. In contrast, the Experienced group not only felt less stress and exhibited more capacity to recover, but their stress and recovery levels remained stable and were less influenced by fasting.

Compared to the Experienced group, the Newbie group reported higher levels of physical and psychological stress, especially at FT-6, when their general stress (Figure 6A; *M ± SD_Experienced_* = 3 ± 0.00, *M ± SD_Newbie_* = 5.43 ± 2.64, Mann-Whitney U = 6, *p* = 0.035, Cohen’s *d* = 1.48), emotional stress (Figure 6B; *M ± SD_Experienced_* = 2 ± 0.00, *M ± SD_Newbie_* = 3.86 ± 1.77, Mann-Whitney U = 6, *p* = 0.035, Cohen’s *d* = 1.48), and fatigue (Figure 6C; *M ± SD_Experienced_* = 6 ± 2.53, *M ± SD_Newbie_* = 10.29 ± 3.73, Mann-Whitney U = 6, *p* = 0.035, Cohen’s *d* = 1.48) were significantly higher. Although the greatest difference between the groups was observed at FT-6, the highest score for the inexperienced participants overall for the stress subscales was measured at FT-3 (Figures 6A,B,E–H).

For the Experienced group, most of their stress scores decreased during and after fasting, and their scores for somatic complaints slightly increased during fasting but rapidly decreased when they had access to food (Figure 6G).

**Figure 7:**
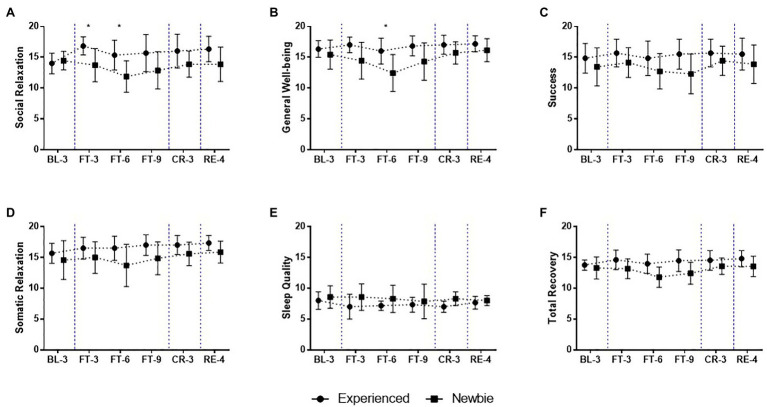
Mean and SD of subscales of Recovery scores over the 22-day experiment for the Experienced and Newbie groups. **(A-F)** The scores of the subscales related to the stress in the recovery-stress questionnaires (RESTQ). Symbols represent mean scores for the two groups, and error bars represent the SD of the mean. ^*^p < 0.05 indicates significant differences between the two groups at a given test point. Blue dashed lines indicate the different sessions.

Recovery capability for the Newbies was also negatively affected by fasting. During the Complete Fasting session, these participants reported lower levels of social relaxation (Figure 7A; FT-3: *M ± SD_Experienced_* = 16.83 ± 1.47, *M ± SD_Inexperienced_* = 13.71 ± 2.69, Mann-Whitney U = 6.5, *p* = 0.035, Cohen’s *d* = 1.48; FT-6: *M ± SD_Experienced_* = 15.33 ± 2.42, *M ± SD_Inexperienced_* = 11.86 ± 2.54, Mann-Whitney U = 6.5, *p* = 0.035, Cohen’s *d* = 1.48) and general well-being (Figure 7B; FT-6: *M ± SD _Experienced_* = 16 ± 2.10, *M ± SD_Newbie_* = 12.43 ± 2.99, Mann-Whitney U = 6.5, *p* = 0.035, Cohen’s *d* = 1.48). This result indicates that the Newbies felt less pleasure in response to social contact. In contrast, the Experienced group reported high scores for recovery resources (Figures 7C,D,F) except for sleep quality (Figure 7E).

## Discussion

The aim of the present study was to investigate the effect of fasting experience on physiological and psychological responses over a 22-day experiment. Consistent with our hypothesis, our participants’ physiological responses (i.e., weight, glucose, and ketone levels) followed the same patterns regardless of their fasting experiences; however, the Newbie group exhibiting markedly more negative psychological responses during the experiment, whereas the Experienced group managed to stabilize their psychological responses, especially during the Complete Fasting session. Specifically, the inexperienced participants reported a greater subjective appetite, more negative mood states, higher levels of stress, higher levels of fatigue, and fewer recovery resources than the Experienced group during the 22-day experiment. This study extends previous fasting experiments by demonstrating that fasting experience is an important buffer against negative feelings induced by a lack of food.

### Similar Patterns of Physiological Responses During Fasting

Our 22-day experiment effectively induced weight loss. As we expected, the pattern of weight loss that occurred was similar in the Newbie and Experienced groups (Figure 2A). Although there was a significant difference in baseline weight between the two groups, this difference was stable and was not affected by fasting time or experience. Consistent with previous studies (Cahill, 2006), the results of the current study demonstrate that ketone bodies could replace glucose as a source of energy during fasting (Figures 2B,C). This process of metabolic change was not affected by whether participants had previous fasting experience.

### Stronger Appetite and Negative Mood for the Newbie Group During Fasting

Intuitively, one would anticipate that food deprivation results in increased appetite, that is, high scores for hunger, high scores for the desire to eat and low scores in fullness. Interestingly, our results suggest that appetite was affected by prior fasting experience (Figure 3). The Experienced group reported a decreased appetite during complete fasting, whereas the Newbie group reported dramatically increased scores for appetite. Previous studies have reported various trends of changes in appetite. For example, in an experiment involving participants following very low-calorie diets over 2 months, participants reported decreased appetite during food deprivation compared to participants who followed a normal balanced diet (Wadden et al., 1987). In 24-h fasting (Cameron et al., 2014) and 4-day acute severe caloric restriction studies (Mars et al., 2006), participants reported increased scores for hunger, the desire to eat, and prospective food consumption and low scores for fullness after the experiments.

The group difference found may be attributable to feelings of hunger combined with feelings of weakness induced by a lack of food and a strong desire to eat (Lowe et al., 2000). For the Experienced group, participants anticipated what they would feel during the study based on their previous fasting experience. This experience may have helped them separate their physiological and psychological sensations of hunger from “thoughts about food.” Inexperienced participants may have failed to distinguish their physical need for food from their psychological desires for food. The failure to distinguish between these two concepts was observed from similar trends of hunger and the desire to eat (Figures 3A,B) during the Complete Fasting session, whereas for the Experienced group, there was a slight increase in the subjective feeling of hunger but a decrease in the desire to eat.

The effect of fasting experience was also found at the peak point of appetite. According to French et al. (2012), hunger and fullness involve both energy balance and affective responses to food-related aspects. In the current study, the highest score for appetite in the Experienced group occurred at FT-6, which is consistent with the new metabolic balance point (Figures 2B,C). From this perspective, the subjective hunger of the Experienced group followed the homeostatic energy balance. For the Newbies, the highest scores for hunger and eating desire were recorded at the beginning of the Complete Fasting session (FT-3), most likely due to affective responses. Without experience, participants may have felt uncertain and stressed about such a novel challenge, and the stress scores for them did peak at FT-3 (Figure 6). We discuss the relationship between appetite and stress further in Section Lower Vitality and Higher Stress for the Newbie Group During Fasting.

The two groups reported similar initial mood states; however, different trends of mood state responses occurred for the Newbie and Experienced groups. Whereas negative mood states did not dramatically change and even decreased in response to a lack of food in the Experienced group, food deprivation increased the Newbie group’s negative mood states from at the beginning of the Complete Fasting session. This group’s negative mood states decreased after FT-6, and they returned to baseline levels after the group received food again. This finding indicates that a lack of fasting experience may lead to intensified food deprivation-induced negative moods. Additionally, a previous study revealed that one-night fasting could enhance fear extinction retention and prevent the return of fear and found this effect to persist for 6 months (Shi et al., 2018), showing the potential influence of fasting on emotion-related regulation. The different intensities and patterns of negative moods found in the two groups provide a potential explanation for inconsistent mood state changes observed in previous fasting studies (e.g., mood enhancement or impairment).

### Lower Vitality and Higher Stress for the Newbie Group During Fasting

Persistent fatigue is a common adverse effect induced by fasting. In the current study, fatigue severity scores increased sharply at the beginning of the Complete Fasting session, especially for the Newbies. Consistent with previous research, this finding demonstrates that energy deficiency can cause subjective fatigue (Roky et al., 2003; Alkandari et al., 2012). Moreover, our results reveal that the Experienced group reported lower scores for fatigue during the Recovery (RE-4) session than during the Baseline (BL-3) session. The results for the Experienced group are in line with the notion that appropriate fasting therapy can serve as a treatment for patients with chronic fatigue syndrome (Masuda et al., 2001). In contrast, for the Newbie group, the scores for fatigue decreased when they had access to food again, but the scores were still higher than the baseline level. This result indicates that the fatigue induced by fasting could be long lasting. We do not have an immediate explanation for why this occurred for the Newbie group only except that the Newbie group was not acquainted with fasting and reported high levels of tiredness and exhaustion; therefore, these individuals needed more time to recover from intense fatigue induced by fasting.

We interestingly found the Experienced group to show slightly low scores for the Response to Rest/Sleep subscale for fatigue (Figure 5D). This result indicates that the Experienced group could not alleviate their fatigue through sleep or rest. The Experienced group may not have needed sleep and rest to lessen their fatigue because they did not experience intense fatigue while fasting. However, this may be unlikely given that the need for sleep to reduce fatigue differs from the belief that sleep/rest could help one recover from fatigue. As an alternative explanation, the Experienced group may not have been able recover from fatigue through sleep or rest. This explanation is supported by the sleep quality subscale of the RESTQ (Figure 7E); the results reveal that the Experienced group reported lower sleep quality than the Newbie group. Because participants from the Experienced group did not have good sleep during the experiment, it is understandable that they could not reduce their fatigue through sleep or rest. However, our results do not allow us to infer the reasons for the poor sleep quality observed in the Experienced group, which requires further research.

The acute fasting experiment was a stressful event for the participants. It is important that individuals are able to balance stress with their own recovery resources, and recovery-stress balance is critical for individual health, well-being, and performance and are also related to participants’ vitality states (Nicolas et al., 2019). Overall, the increase in subjective stress and decrease in subjective recovery observed suggest maladaptive adjustment at the start of the Complete Fasting session. Compared to the Newbie group, the Experienced group showed a better stress-recovery balance; that is, they had significantly lower scores for stress and higher scores for recovery resources (Total stress: Figure 6H; Total recovery: Figure 7F). The differences in perceptions of subjective stress and recovery found for the two groups indicate that fasting was not as strong of a stressor for the Experienced group as it was for the Newbies. In other words, fasting triggered stress responses in all of the participants, but fasting may have triggered overactive stress responses in the Newbies with no first-hand experience.

The highest level of subjective stress was observed at the beginning of the Complete Fasting session (FT-3) for the Newbie group and at the middle of the Complete Fasting session for the Experienced group (FT-6). As we mention above (see Discussion “Stronger Appetite and Negative Mood for the Newbie Group During Fasting”), the perceived stress levels of the two groups coincided with their subjective feelings of appetite (Figures 3, 6). Additionally, while we are unable to infer causality from subjective feelings of stress and appetite, the Experienced group showed low levels of stress and hunger and desires to eat, while the Newbies reported high levels of these psychological responses. One explanation for these findings is rooted in the stress-induced eating hypothesis, which indicates that subjective feelings of hunger increase in intensity as perceived stress increases (Huh et al., 2015). Through concurrent ratings of perceived stress and hunger collected over 7 days *via* hourly text messaging assessments and real-time eating records, researchers have demonstrated a generally positive stress–hunger relationship in young adults (Huh et al., 2015). The “higher stress, higher appetite” result of the current study empirically confirms the positive relationship between subjective stress and appetite (Sarker et al., 2013). More importantly, the current study indicates that prior fasting experience may help attenuate responses to stressors (e.g., current fasting).

### Implications

Although a large number of fasting studies have tested physiological and psychological reactions, they have mostly focused on various fasting protocols for fasting/non-fasting or patient/healthy participants. The current study highlights the potential effect of prior fasting experience, which may provide insights for future training and fundamental research on fasting.

On a theoretical level, studying fasting experience can contribute to general research on fasting. As shown by the present study, experiences can influence how people feel while fasting, serving as a crucial factor when selecting participants for future studies. Moreover, the basic physiological indexes did not show differences for fasting based on experience, and future studies could investigate more physiological indexes and examine to what extent our fasting-induced mechanisms are affected by fasting experience.

On an applied level, the current study indicates the important role of fasting experience. Note that appropriate fasting protocols should be cautiously chosen based on individual health conditions and doctors’ advice in daily life, and long-term complete fasting should be conducted with intensive medical supervision and in a laboratory environment to prevent any adverse effects. Our study still indicates that fasting training can be applied in preparation for emergency situations in some occupations. For example, in future space exploration missions it could be effective to allow crew members to obtain a first-hand experience with food deprivation during training to help them understand that following fasting protocols is feasible and to know how fasting feels. Fasting training may also be extended to ground investigations, such as polar explorations and mountaineering expeditions.

### Limitations

Our interpretations of the results should be considered within the experimental limitations of the study. One limitation of this study concerns its small sample size, and our results thus should be interpreted with caution. Although the significant effects observed in the current preliminary experiment suggest adequate power for the statistical analysis approach, we cannot safely rule out individual differences. From our preliminary experiment, which showed 10 days of complete fasting to be feasible, we could recruit more participants and ask more questions about previous fasting experiences (e.g., how many times individuals have practiced fasting before, which kinds of fasting protocols have been adopted, and fasting durations). In this way, we can develop a stronger understanding of how fasting experiences influence participants’ psychological and physiological responses to food deprivation.

The motivations for fasting may also impact participants’ psychological responses. It is possible that participants with weight concerns tend to have more positive feelings about fasting, and they are more likely to have practiced fasting before. This may explain why the average weight of the Experienced group was higher than that of the Newbies in the current study. However, it is unlikely that differences in psychological responses merely relied on weight differences because the trajectories of psychological states were not in parallel with the decrease in weight. Because, we cannot fully rule out the effect of weight, screening and matching for weight between groups will be necessary in future studies. Additionally, future research should measure motivations for participating in fasting research, for example, goals participants want to achieve through fasting experiments and how they perceive fasting (e.g., as a form of therapy, cost-free weight management measure, or religious practice).

Another concern relates to the various strategies of coping with fasting. For example, a previous study showed that increased self-regulation through mindfulness practice may assist people in adhering to weight management (Spadaro et al., 2018), indicating the critical role of psychological meditations. Additionally, people regularly engaged in fasting may develop their own coping strategies; for example, athletes make create own training schedules to avoid adverse effects during Ramadan fasting, which may lead to reduced potential negative feelings (Roy et al., 2011). Hence, in future research, open-ended questions that measure psychological coping strategies can provide more information about how individuals adjust fasting.

## Conclusion

The overarching aim of the current study was to examine the effect of previous fasting experience on participants’ physiological and psychological responses over a 22-day period. As hypothesized, the Newbie group showed stronger negative feelings during fasting, and their feelings fluctuated over the fasting period, whereas the Experienced group showed stable psychological responses. The physiological responses did not differ based on previous fasting experience. Again, with the present study, we do not suggest or advocate people regularly practicing long-term complete fasting, especially without doctoral advices and intensive medical supervision. Our novel results provide rich insights into how fasting experience influences mental states related to food deprivation, which should be considered in future fasting and eating behavior research.

## Data Availability Statement

The raw data supporting the conclusions of this article will be made available by the authors, without undue reservation.

## Ethics Statement

The studies involving human participants were reviewed and approved by Ethics Committee of Space Institute of Southern China. The patients/participants provided their written informed consent to participate in this study.

## Author Contributions

QM and CY contributed to executing the experiments, analyzing the data, interpreting the results, and writing the original draft. CY, RW, ZD, and YL contributed to framing the study theoretically and designing. MW and WL contributed to executing the experiments. All authors contributed to the article and approved the submitted version.

### Conflict of Interest

The authors declare that the research was conducted in the absence of any commercial or financial relationships that could be construed as a potential conflict of interest.

## Footnotes

^1^http://psychometrica.de/effect_size.html
