# Supplementary material for: How Experiences Affect Psychological Responses During Supervised Fasting: A Preliminary Study
Source: Front Psychol. 2021 May 19;12:651760. doi: 10.3389/fpsyg.2021.651760 (PMC8170085; doi:10.3389/fpsyg.2021.651760)
Supplement: Supplementary file 1 [file Table_1.DOCX]

**Table S1** Effect of Fasting experience on physiological responses to over the 22-days experiment

| Variables | Groups | BL-3 | FT-3 | FT-6 | FT-9 | CR-3 | RE-4 |
| --- | --- | --- | --- | --- | --- | --- | --- |
| Weight (kg) | Experienced | **79.63±9.26** | **76.70±8.54** | **74.10±8.74** | **72.40±8.55** | **71.43±7.98** | **75.97±8.35** |
|  | Newbie | **65.36±11.08** | **63.43±11.12** | **60.80±10.63** | **59.46±10.09** | **58.61±9.86** | **62.09±9.04** |
|  | Mann-Whitney U | **6** | **6** | **6** | **6** | **6** | **5** |
|  | *p (Cohen’s d)* | **0.035 (1.48)**  **BF_-0_: 2.56** | **0.035 (1.48)**  **BF_-0_: 2.82** | **0.035 (1.48)**  **BF_-0_: 2.84** | **0.035 (1.48)**  **BF_-0_: 2.63** | **0.035 (1.48)**  **BF_-0_: 3.26** | **0.022 (1.65)**  **BF_-0_: 2.93** |
| Glucose | Experienced | 5.1±0.71 | 3.29±0.74 | 2.90±0.47 | 3.80±0.88 | 4.72±0.73 | 4.75±0.58 |
|  | Newbie | 4.98±0.41 | 3.26±0.56 | 2.76±0.32 | 3.72±0.55 | 4.85±0.31 | 4.47±0.20 |
|  | Mann-Whitney U | 18 | 21 | 18 | 20 | 19 | 18 |
|  | *p* | 0.73 | 1.00 | 0.73 | 0.95 | 0.84 | 0.73 |
| Ketone body | Experienced | 0.18±0.04 | 2.25±0.92 | 5.55±1.34 | 5.87±0.99 | 3.47±1.30 | 0.2±0.00 |
|  | Newbie | 0.17±0.05 | 2.73±1.08 | 5.54±0.83 | 5.4±0.85 | 3.03±1.60 | 0.2±0.00 |
|  | Mann-Whitney U | 18.5 | 15 | 18.5 | 13 | 17 | 21 |
|  | *p* | 0.73 | 0.45 | 0.73 | 0.30 | 0.63 | 1.00 |

**Note:** *p* indicates the exact significance; **Bold:** significant results in Independent Samples Mann-Whitney U Test; Cohen's d effect size was further calculated with online calculator for the significant results (<http://psychometrica.de/effect_size.html>). BF-0 factor is calculated by JASP0.14.1.0, indicated that the data are x times more likely under the alternative hypothesis (the Experienced Group had higher scores than the Newbie Group) than the null hypothesis (no differences between two groups).

**Table S2** Effect of Fasting experience on subjective appetite over the 22-days experiment assessed by Visual Analogous Scale

| Variables | Group | BL-3 | FT-3 | FT-6 | FT-9 | CR-3 | RE-4 |
| --- | --- | --- | --- | --- | --- | --- | --- |
| Hungriness | Experienced | 1.83±2.40 | **2.33±1.86** | **3.17±1.83** | **2.5±1.76** | 1.67±1.75 | 1.17±1.94 |
|  | Newbie | 1.86±2.79 | **6.43±2.23** | **6.50±1.87** | **5.71±2.36** | 2.57±2.14 | 2.43±2.07 |
|  | Mann-Whitney U | 20.5 | **3** | **3.5** | **6.5** | 16 | 13 |
|  | *p (Cohen’s d)* | 0.95 | **0.008 (2.04)**  **BF_+0_: 4.67** | **0.015 (1.93)**  **BF_+0_: 4.24** | **0.035 (1.40)**  **BF_+0_: 2.80** | 0.53 | 0.30 |
| Desire of Eating | Experienced | 3.33±2.50 | **1.33±1.03** | 2.17±1.47 | 1.67±1.51 | 3.50±2.17 | 3.67±2.50 |
|  | Newbie | 3.14±2.41 | **5.29±2.36** | 5.29±3.04 | 4.71±2.98 | 4.43±2.51 | 5.71±2.93 |
|  | Mann-Whitney U | 19.5 | **1.5** | 8 | 8 | 17 | 11.5 |
|  | *p (Cohen’s d)* | 0.84 | **0.002 (2.43)**  **BF_+0_: 6.01** | 0.07 | 0.07 | 0.63 | 0.18 |
| Fullness | Experienced | 4.40±1.52 | 4.17±2.79 | 3.5±2.81 | 3.67±3.61 | 6.50±3.01 | 7.17±1.72 |
|  | Newbie | 4.43±3.36 | 1.14±1.07 | 1.29±0.95 | 1±0.82 | 5±2.83 | 6.29±1.11 |
|  | Mann-Whitney U | 17 | 7.5 | 12 | 13 | 11.5 | 13.5 |
|  | *p* | 1 | 0.05 | 0.23 | 0.30 | 0.18 | 0.30 |

**Note:** *p* indicates the exact significance; **Bold:** significant results in Independent Samples Mann-Whitney U Test; *Cohen's d* effect size was further calculated with online calculator for the significant results (<http://psychometrica.de/effect_size.html>). BF+0 factor is calculated by JASP0.14.1.0, indicated that the data are x times more likely under the alternative hypothesis (the Newbie Group had higher scores than the Experienced Group) than the null hypothesis (no differences between two groups).

**Table S3** Effect of Fasting experience on mood states over the 22-days experiment assessed by Profile of Mood States

| Variables | Group | BL-3 | FT-3 | FT-6 | FT-9 | CR-3 | RE-4 |
| --- | --- | --- | --- | --- | --- | --- | --- |
| Tension-Anxiety | Experienced | 10.5±1.64 | 9.67±1.03 | 10±0.89 | 9.67±1.21 | 8±0.00 | 9.17±0.41 |
|  | Newbie | 12.29±3.91 | 14.29±5.31 | 13.57±5 | 12.71±3.25 | 10.14±3.76 | 10.71±2.06 |
|  | Mann-Whitney U | 16 | 9 | 11 | 7 | 12 | 11 |
|  | *p* | 0.54 | 0.10 | 0.18 | 0.05 | 0.23 | 0.18 |
| Depression-Dejection | Experienced | 16.33±2.42 | 16±1.67 | **15.17±0.41** | 15.17±0.41 | 15.17±0.41 | 15±0 |
|  | Newbie | 17.29±3.35 | 19.14±4.88 | **19.14±4.38** | 18.43±5.16 | 16.57±2.44 | 16.57±2.70 |
|  | Mann-Whitney U | 18 | 13 | **4.5** | 14 | 14.5 | 15 |
|  | *p (Cohen’s d)* | 0.73 | 0.30 | **0.014 (1.73)**  **BF_+0_: 3.30** | 0.37 | 0.37 | 0.45 |
| Anger-Hostility | Experienced | 16±2.97 | 14.67±1.75 | 14.83±1.60 | 14.67±1.03 | 14.50±1.64 | 14.67±1.51 |
|  | Newbie | 15.29±4.42 | 14.43±3.15 | 15.14±3.53 | 14.14±3.39 | 13.14±1.86 | 13.29±1.60 |
|  | Mann-Whitney U | 15.5 | 18.5 | 19. | 12 | 11.5 | 11 |
|  | *p* | 0.45 | 0.73 | 0.84 | 0.23 | 0.18 | 0.18 |
| Vigor-Activity | Experienced | 31.83±4.31 | 31±6.54 | 27.17±8.28 | 28.67±7.00 | 32.50±6.60 | 32.17±6.85 |
|  | Newbie | 29.86±4.41 | 24.71±7.16 | 18.86±5.08 | 23.14±5.98 | 29.57±4.89 | 29.86±5.01 |
|  | Mann-Whitney U | 16.5 | 11 | 8 | 13 | 16 | 16 |
|  | *p* | 0.53 | 0.18 | 0.07 | 0.30 | 0.53 | 0.53 |
| Fatigue-Inertia | Experienced | 10±4.29 | 11.17±4.67 | **14.33±4.63** | 11.50±5.43 | 9.17±5.31 | 8.17±2.86 |
|  | Newbie | 12.86±8.21 | 18.14±9.74 | **25.14±8.91** | 20.86±11.35 | 12.14±6.52 | 9±4.04 |
|  | Mann-Whitney U | 19 | 12 | **6** | 10 | 13.5 | 16 |
|  | *p (Cohen’s d)* | 0.84 | 0.23 | **0.035 (1.48)**  **BF_+0_: 3.19** | 0.14 | 0.30 | 0.53 |
| Confusion-Bewilderment | Experienced | 9.83±2.71 | 8.83±2.13 | **9.33±1.37** | 9.17±2.14 | 7.17±0.98 | 8.5±2.35 |
|  | Newbie | 10.43±4.32 | 13.57±4.83 | **14.14±3.58** | 12.14±4.38 | 8.43±2.37 | 10±2.58 |
|  | Mann-Whitney U | 19.5 | 11.5 | **4** | 12.5 | 13 | 12 |
|  | *p (Cohen’s d)* | 0.84 | 0.18 | **0.014 (****1.82)**  **BF_+0_: 4.09** | 0.23 | 0.30 | 0.23 |
| Total Mood Disturbance | Experienced | 130.83±14.06 | 129.33±12.23 | **136.50±12.13** | 131.5±10.37 | 121.50±10.37 | 123.33±9.85 |
|  | Newbie | 138.29±23.31 | 154.86±27.70 | **168.29±22.50** | 155.14±24.88 | 130.86±14.14 | 129.71±10.93 |
|  | Mann-Whitney U | 19 | 11.5 | **6** | 11 | 12 | 13 |
|  | *p (Cohen’s d)* | 0.84 | 0.18 | **0.035 (1.48)**  **BF_+0_: 3.32** | 0.18 | 0.23 | 0.30 |

**Note:** *p* indicates the exact significance; **Bold:** significant results in Independent Samples Mann-Whitney U Test; *Cohen's d* effect size was further calculated with online calculator for the significant results (<http://psychometrica.de/effect_size.html>). BF+0 factor is calculated by JASP0.14.1.0, indicated that the data are x times more likely under the alternative hypothesis (the Newbie Group had higher scores than the Experienced Group) than the null hypothesis (no differences between two groups).

**Table S4** Effect of Fasting experience on fatigue over the 22-days experiment assessed by Fatigue Assessment Instrument

| Variables | Group | BL-3 | FT-3 | FT-6 | FT-9 | CR-3 | RE-4 |
| --- | --- | --- | --- | --- | --- | --- | --- |
| Severity | Experienced | 3.27±0.79 | 3.80±0.56 | **3.61±0.88** | 3.56±0.98 | **2.97±1.51** | 2.59±1.57 |
|  | Newbie | 3.06±1.17 | 4.19±0.98 | **5.05±0.73** | 4.62±1.55 | **4.61±1** | 4.16±1.45 |
|  | Mann-Whitney U | 20.5 | 18 | **4** | 12 | **6.5** | 9.5 |
|  | *p (Cohen’s d)* | 0.95 | 0.73 | **0.014 (1.82)**  **BF_+0_: 4.11** | 0.23 | **0.035 (1.40)**  **BF_+0_: 3.37** | 0.10 |
| Situation-Specificity | Experienced | 3.27±0.79 | 3.78±1.35 | 3.86±1.16 | 3.64±1.55 | 3.69±1.82 | 3±1.83 |
|  | Newbie | 3.57±1.62 | 4.26±0.95 | 4.36±0.82 | 4.62±1.22 | 4.57±0.74 | 4.31±0.78 |
|  | Mann-Whitney U | 18.5 | 15 | 14.5 | 13 | 13.5 | 15 |
|  | *p* | 0.73 | 0.45 | 0.37 | 0.30 | 0.30 | 0.45 |
| Psychological Consequences | Experienced | 4.67±0.73 | 4.22±1.59 | 3.56±1.56 | 3.83±1.87 | 3.22±1.81 | 3.06±2.04 |
|  | Newbie | 3.48±2.09 | 5.10±1.05 | 5.33±1.47 | 3.81±1.89 | 4.86±1.55 | 4.90±2.10 |
|  | Mann-Whitney U | 13 | 12.5 | 8.5 | 20.5 | 9 | 10 |
|  | *p* | 0.30 | 0.23 | 0.07 | 0.95 | 0.10 | 0.14 |
| Responds to Rest/Sleep | Experienced | 5.67±1.25 | 5.92±1.39 | 6±0.63 | 6.25±0.76 | 5.58±2.06 | 4.83±1.94 |
|  | Newbie | 5.79±2.18 | 6.71±0.49 | 6.64±0.75 | 5.64±1.35 | 6.36±0.75 | 6.36±0.75 |
|  | Mann-Whitney U | 16.5 | 13.5 | 9 | 16 | 17 | 10 |
|  | *p* | 0.53 | 0.30 | 0.10 | 0.53 | 0.63 | 0.14 |

**Note:** *p* indicates the exact significance; **Bold:** significant results in Independent Samples Mann-Whitney U Test; *Cohen's d* effect size was further calculated with online calculator for the significant results (<http://psychometrica.de/effect_size.html>). BF+0 factor is calculated by JASP0.14.1.0, indicated that the data are x times more likely under the alternative hypothesis (the Newbie Group had higher scores than the Experienced Group) than the null hypothesis (no differences between two groups).

**Table S5** Effect of Fasting experience on stress and recovery state over the 22-days experiment assessed by the Recovery-Stress Questionnaire

| Variables | Group | BL-3 | FT-3 | FT-6 | FT-9 | CR-3 | RE-4 |
| --- | --- | --- | --- | --- | --- | --- | --- |
| General Stress | Experienced | 3.67±1.21 | 3.33±0.52 | **3±0.00** | 3.17±0.41 | 3±0 | 3±0 |
|  | Newbie | 4.14±1.22 | 5.43±2.57 | **5.43±2.64** | 4.86±1.86 | 4.43±1.81 | 3.29±0.76 |
|  | Mann-Whitney U | 16 | 12 | **6** | 10.5 | 12 | 18 |
|  | *p (Cohen’s d)* | 0.53 | 0.23 | **0.035 (1.48)**  **--** | 0.14 | 0.23 | 0.73 |
| Emotional Stress | Experienced | 2.5±0.55 | 2.5±1.22 | **2±0.00** | 2±0.00 | 2±0 | 2±0 |
|  | Newbie | 3.86±2.34 | 4.29±1.80 | **3.86±1.77** | 3.29±1.38 | 2.86±1.07 | 2.29±0.49 |
|  | Mann-Whitney U | 15 | 9 | **6** | 9 | 12 | 15 |
|  | *p (Cohen’s d)* | 0.45 | 0.10 | **0.035 (1.48)**  **--** | 0.10 | 0.23 | 0.45 |
| Social Stress | Experienced | 3.83±1.17 | 3.5±0.84 | 3.33±0.82 | 3.17±0.41 | 3.17±0.41 | 3±0 |
|  | Newbie | 4.43±1.99 | 4.86±1.95 | 5.43±2.44 | 4.86±2.41 | 4.71±1.98 | 3.71±1.25 |
|  | Mann-Whitney U | 19 | 12.5 | 10.5 | 14 | 11 | 15 |
|  | *p* | 0.84 | 0.23 | 0.14 | 0.37 | 0.18 | 0.45 |
| Conflicts/Pressure | Experienced | 10.67±2.80 | 7.83±3.43 | 8.83±2.56 | 8.5±3.15 | 8±3.46 | 8±2.97 |
|  | Newbie | 8.57±3.87 | 10.29±3.20 | 9.57±2.57 | 9.29±2.14 | 9.29±2.29 | 9±2.38 |
|  | Mann-Whitney U | 13.5 | 11.5 | 17.5 | 16.5 | 15.5 | 17 |
|  | *p* | 0.30 | 0.18 | 0.63 | 0.53 | 0.45 | 0.63 |
| Fatigue | Experienced | 7±3.10 | 5.83±1.17 | **6±2.53** | 6±2.76 | 5.67±2.42 | 4.17±1.60 |
|  | Newbie | 6.86±4.56 | 9±4.65 | **10.29±3.73** | 9±4 | 5.43±2.37 | 4.71±2.06 |
|  | Mann-Whitney U | 17 | 14.5 | **6** | 10.5 | 19.5 | 18 |
|  | *p (Cohen’s d)* | 0.63 | 0.37 | **0.035 (1.48)**  **BF_+0_: 3.09** | 0.14 | 0.84 | 0.73 |
| Lack of Energy | Experienced | 7.33±2.50 | 6±2.19 | 7.17±2.40 | 6.17±1.94 | 5.33±1.86 | 5±1.67 |
|  | Newbie | 6.29±2.56 | 9.14±3.98 | 9.14±3.02 | 8.14±4.95 | 6.14±2.41 | 4.43±1.81 |
|  | Mann-Whitney U | 14.5 | 10.5 | 9.5 | 16 | 17 | 16 |
|  | *p* | 0.37 | 0.14 | 0.10 | 0.53 | 0.63 | 0.53 |
| Somatic Complaints | Experienced | 4.5±1.52 | 4.83±2.56 | 5.5±2.74 | 5.67±2.80 | 4±2 | 3±0 |
|  | Newbie | 4.71±1.89 | 7.57±3.10 | 7.29±3.35 | 6.71±3.68 | 4.43±1.51 | 3.57±0.79 |
|  | Mann-Whitney U | 20.5 | 10 | 14 | 18.5 | 16.5 | 12 |
|  | *p* | 0.85 | 0.14 | 0.37 | 0.73 | 0.53 | 0.23 |
| Total stress | Experienced | 5.64±0.69 | 4.83±0.91 | 5.12±0.73 | 4.95±0.83 | 4.45±0.60 | 4.02±0.58 |
|  | Newbie | 5.55±2.26 | 7.22±2.51 | 7.29±2.08 | 6.59±2.49 | 5.33±1.64 | 4.43±1.08 |
|  | Mann-Whitney U | 19.5 | 11 | 10 | 16 | 17.5 | 16.5 |
|  | *p* | 0.84 | 0.18 | 0.14 | 0.53 | 0.63 | 0.53 |
| Success | Experienced | 14.83±2.40 | 15.67±2.25 | 14.83±2.79 | 15.50±2.43 | 15.67±2.25 | 15.50±2.59 |
|  | Newbie | 13.43±3.10 | 14.14±2.41 | 12.71±2.87 | 12.29±3.25 | 14.43±2.37 | 13.86±3.13 |
|  | Mann-Whitney U | 15 | 15 | 11.5 | 8 | 14 | 14.5 |
|  | *p* | 0.45 | 0.45 | 0.18 | 0.07 | 0.37 | 0.37 |
| Social Relaxation | Experienced | 14±1.67 | **16.83±1.47** | **15.33±2.42** | 15.67±3.01 | 16±2.76 | 16.33±2.07 |
|  | Newbie | 14.43±1.51 | **13.71±2.69** | **11.86±2.54** | 12.86±3.02 | 13.86±2.12 | 13.86±2.79 |
|  | Mann-Whitney U | 17.5 | **6.5** | **6.5** | 10 | 9 | 9.5 |
|  | *p (Cohen’s d)* | 0.63 | **0.035 (1.48)**  **BF_-0_: 4.46** | **0.035 (1.48)**  **BF_-0_: 3.01** | 0.14 | 0.10 | 0.10 |
| Somatic Relaxation | Experienced | 15.67±1.63 | 16.5±1.76 | 16.5±1.97 | 17±1.67 | 17±1.55 | 17.33±1.21 |
|  | Newbie | 14.57±3.15 | 15±2.58 | 13.71±3.45 | 14.86±2.67 | 15.57±1.90 | 15.86±1.77 |
|  | Mann-Whitney U | 17 | 13.5 | 9.5 | 11 | 12 | 11 |
|  | *p* | 0.63 | 0.30 | 0.10 | 0.18 | 0.23 | 0.18 |
| General Well-being | Experienced | 16.33±1.37 | 17±1.26 | **16±2.10** | 16.83±1.60 | 17±1.55 | 17.17±1.33 |
|  | Newbie | 15.43±2.37 | 14.43±2.99 | **12.43±2.99** | 14.29±3.04 | 15.71±1.80 | 16.14±1.86 |
|  | Mann-Whitney U | 16 | 10 | **6.5** | 11 | 13 | 14 |
|  | *p (Cohen’s d)* | 0.53 | 0.14 | **0.035 (1.48)**  **BF_-0_: 3.19** | 0.18 | 0.30 | 0.37 |
| Sleep Quality | Experienced | 8±1.41 | 7±2 | 7.17±0.75 | 7.33±1.21 | 7±0.89 | 7.67±1.03 |
|  | Newbie | 8.57±1.81 | 8.57±2.15 | 8.29±2.21 | 7.86±2.79 | 8.29±1.11 | 8±0.82 |
|  | Mann-Whitney U | 18.5 | 12.5 | 14 | 14 | 8 | 17.5 |
|  | *p* | 0.73 | 0.23 | 0.37 | 0.37 | 0.07 | 0.63 |
| Total recovery | Experienced | 13.77±0.82 | 14.6±1.58 | 13.97±1.56 | 14.47±1.74 | 15.53±1.59 | 14.80±1.31 |
|  | Newbie | 13.29±1.77 | 13.17±1.61 | 11.80±1.67 | 12.43±1.79 | 13.57±1.33 | 13.54±1.65 |
|  | Mann-Whitney U | 18 | 10 | 8 | 9.5 | 13 | 9 |
|  | *p* | 0.73 | 0.14 | 0.07 | 0.10 | 0.30 | 0.10 |

**Note:** *p* indicates the exact significance; **Bold:** significant results in Independent Samples Mann-Whitney U Test; *Cohen's d* effect size was further calculated with online calculator for the significant results (<http://psychometrica.de/effect_size.html>). BF+0 factor is calculated by JASP0.14.1.0, indicated that the data are x times more likely under the alternative hypothesis (the Newbie Group had higher stress states than the Experienced Group) than the null hypothesis (no differences between two groups). Also, BF-0 factor is calculated by JASP0.14.1.0, indicated that the data are x times more likely under the alternative hypothesis (the Experienced Group had higher scores than the Newbie Group) than the null hypothesis (no differences between two groups).
